# Identification of Stage IIIC/IV EGFR-Mutated Non-Small Cell Lung Cancer Populations Sensitive to Targeted Therapy Based on a PET/CT Radiomics Risk Model

**DOI:** 10.3389/fonc.2021.721318

**Published:** 2021-11-02

**Authors:** Dan Shao, Dongyang Du, Haiping Liu, Jieqin Lv, You Cheng, Hao Zhang, Wenbing Lv, Shuxia Wang, Lijun Lu

**Affiliations:** ^1^ Department of Positron Emission Tomography (PET) Center, Guangdong Provincial People’s Hospital, Guangdong Academy of Medical Sciences, Guangzhou, China; ^2^ School of Biomedical Engineering and Guangdong Provincial Key Laboratory of Medical Image Processing, Southern Medical University, Guangzhou, China; ^3^ Department of Positron Emission Tomography/Computed Tomography (PET/CT) Center, The First Affiliated Hospital of Guangzhou Medical University, Guangzhou, China

**Keywords:** lung cancer, PET/CT, radiomics, stratification of progression risk, progression-free survival

## Abstract

**Objectives:**

This project aimed to construct an individualized PET/CT prognostic biomarker to accurately quantify the progression risk of patients with stage IIIC-IV epidermal growth factor receptor (EGFR)-mutated Non-small cell lung cancer (NSCLC) after first-line first and second generation EGFR- tyrosine kinase inhibitor (TKI) drug therapy and identify the first and second generation EGFR-TKI treatment-sensitive population.

**Methods:**

A total of 250 patients with stage IIIC-IV EGFR-mutated NSCLC underwent first-line first and second generation EGFR-TKI drug therapy were included from two institutions (140 patients in training cohort; 60 patients in internal validation cohort, and 50 patients in external validation cohort). 1037 3D radiomics features were extracted to quantify the phenotypic characteristics of the tumor region in PET and CT images, respectively. A four-step feature selection method was performed to enable derivation of stable and effective signature in the training cohort. According to the median value of radiomics signature score (Rad-score), patients were divided into low- and high-risk groups. The progression-free survival (PFS) behaviors of the two subgroups were compared by Kaplan–Meier survival analysis.

**Results:**

Our results shown that higher Rad-scores were significantly associated with worse PFS in the training (*p* < 0.0001), internal validation (*p* = 0.0153), and external validation (*p* = 0.0006) cohorts. Rad-score can effectively identify patients with a high risk of rapid progression. The Kaplan–Meier survival curves of the three cohorts present significant differences in PFS between the stratified slow and rapid progression subgroups.

**Conclusion:**

The PET/CT-derived Rad-score can realize the precise quantitative stratification of progression risk after first-line first and second generation EGFR-TKI drug therapy for NSCLC and identify EGFR-mutated NSCLC populations sensitive to targeted therapy, which might help to provide precise treatment options for NSCLC.

## Introduction

Non-small cell lung cancer (NSCLC) is the leading cause of cancer death worldwide (1). The onset of NSCLC is insidious, and most patients present at an advanced stage. For advanced NSCLC, platinum-based combination chemotherapy has reached a bottleneck, with suboptimal efficacy. In recent years, targeted therapy has achieved remarkable results in cancer treatment, showing great promise in the treatment of a variety of advanced tumors. In patients with NSCLC, especially in East Asian populations, the mutation rate of human epidermal growth factor receptor (EGFR) is as high as 40%~55% (2). Therefore, researchers have developed small-molecule tyrosine kinase inhibitor (TKI) drugs that target EGFR in EGFR gene mutation therapy, which have subsequently been found to prolong progression-free survival (PFS) in such patients when compared with chemotherapy in clinical application (3), extend the median PFS of patients to 9~11 months (4).

However, not all patients can benefit from them. In the clinical application, it is found that for a large part of NSCLC patients with Phase IIIC/IV EGFR mutation after the first-line EGFR-TKI treatment the disease progressed rapidly and they could not benefit from the treatment. The third generation of TKI Osimertinib can simultaneously suppress the EGFR-TKI-sensitive gene and T790M, the drug resistance gene of the first and second generation of TKIs, and extend the median PFS of patients. However, not all patients need the treatment of Osimertinib. For some patients, the treatment effect of the first and second generation of TKIs is very good (5). Therefore, how to screen the patients suitable for the treatment of the first and second generation of TKIs is particularly important. The progress of patients receiving the clinical treatment after the treatment of the first and second generation of EGFR-TKIs was accurately quantified and evaluated, thus recognizing the population not sensitive to the treatment, guiding the clinical decision, making the corresponding treatment and follow-up plan, and improving prognosis. It has an important clinical value.

In 2012, Lambin et al. (6) first proposed the concept of radiomics, that is, the high-throughput extraction of image information from medical images and deep-seated mining and analysis of massive image data to reveal the pathophysiological characteristics of diseases (such as tumor heterogeneity) and provide the most accurate decision support for disease diagnosis, prognosis prediction and precision treatment (7–10). In the past, by using the technical means of radiomics in medical imaging, such as computed tomography (CT), magnetic resonance imaging (MRI), ultrasound and positron emission tomography (PET), multiple research teams have revealed the associations between microscopic image information and tumor genotyping, tumor treatment efficacy and prognosis through deep-seated information mining and analysis (11).

^18^F-fluordeoxyglucos (^18^F-FDG) PET/CT radiomics analysis can extract additional layers of information on tumor heterogeneity, such as biological metabolism information and anatomical characteristic (12). Tumor cell glucose metabolism produces different genetic variations during growth and treatment, so the glucose metabolism at different sites in the same tumor lesion shows significant heterogeneity (13). ^18^F-FDG PET, which can reflect the glucose metabolic intensity information of tumor lesions, has played an important role in clinical application for NSCLC (14–17). PET/CT radiomics features can be used as an individualized prognostic biomarker for patients with NSCLC (18–20). Kang F et al. successfully applied the radiomics in the diagnosis and differential diagnosis of lung cancer to reduce the false negative rate (21). Zhang J et al. showed that PET/CT radiomics model could recognize the mutation type of EGFR (22).

In recent years, PET/CT radiomics has been used to quantify the risk of progression of patients with lung cancer after treatment, gastric cancer, and glioblastoma (14–16). However, few studies based on PET/CT radiomics to accurately predict the risk of progression after first and second generation of EGFR-TKI therapy in stage IIIC/IV EGFR-mutated NSCLC patients (23). To address the clinical challenges that the progression risk of patients with stage IIIC-IV EGFR-mutated NSCLC is difficult to accurately quantify and stratify after first-line first and second generation of EGFR-TKI targeted drug therapy, we aimed to construct an individualized prognostic biomarker to accurately quantify the progression risk of patients with stage IIIC-IV EGFR-mutated NSCLC after first-line first and second generation of EGFR-TKI drug therapy and identify the first and second generation of EGFR-TKI treatment-sensitive population, which is expected to aid clinicians in treatment decision-making.

## Materials and Methods

This retrospective, two-center study was approved by our Institutional Review Board. The requirement for written consent was waived by the board.

### Patients

This 2-center retrospective study was conducted jointly by two independent departments. This study was conducted in accordance with the Declaration of Helsinki. Our Institutional Review Board approved this retrospective study and waived the requirement for informed consent from the patients. Before treatment, all patients successively underwent clinical examination, PET/CT scan, blood tests, and pathological examination. The inclusion criteria were as follows: 1. age 18 and older, stage IIIC-IV NSCLC according to the TNM classification system of the American Joint Committee on Cancer; 2. EGFR mutation; 3. TKI group patients were treated with first-line first and second generation EGFR-TKI according to the criteria established by the National Comprehensive Cancer Network (NCCN) until disease progression, with doses appropriately reduced if severe adverse events occurred; and 4. pretherapy PET/CT was acquired two weeks before the initiation of EGFR-TKI therapy. The exclusion criteria were as follows: 1. patients with a history of a second neoplasm; 2. patients with a history of anticancer therapy or surgical therapy; and 3. HIV-positive patients. The clinicopathological information included epidemiological information and characteristics related to the risk of progression after treatment, including age, sex, and smoking history, pathological type, TNM stage, performance status (PS) score, EGFR gene mutation type, carcinoembryonic antigen (CEA) value, NSCLC-associated antigen value (CYFRA21-1), and the presence or absence of brain/bone/liver/lung/pleural/adrenal metastases. All of the clinicopathological information were complete record for all eligible patients.

In total, 200 patients were recruited from July 2007 to July 2019 from center 1. These patients were randomized into training (N = 140) and internal validation (N = 60) cohorts. Using the same inclusion criteria, 50 patients who initiated TKI-therapy between June 2008 and June 2018 from center 2 was subsequently accrued. This was used as external validation cohort (N = 50). The follow-up interval was 4-6 weeks, and the examinations included clinical physical examination, routine laboratory tests and chest CT or PET/CT. The follow-up duration of this project was 2 years. If no endpoint event (disease progression or death) occurred, the follow-up duration should be at least 3 years. PFS was considered the time from the initiation of EGFR-TKI therapy to the date of confirmed disease progression or death. PFS was censored at the date of death from other causes or the date of the last follow-up visit for progression-free patients.

### PET/CT Scans

PET/CT scans of the training and internal validation cohorts were performed in center 1 using a Sensation Biograph Somatom 16 HR PET/CT machine (SIEMENS, Germany). Scans of external validation cohort were acquired in center 2 on eight-section PET/CT scanner (Discovery ST 8; GE Healthcare, Wisconsin, USA). All patients fasted for at least 6 hours before the PET/CT scan. Only patients with blood glucose levels between 72.0 and 144.0 mg/dL (4.0 - 8.0 mmol/L) were subjected to PET/CT scan. The patients were instructed to lie still in a quiet room for 60 ± 5 minutes after they received an intravenous injection of 0.1-0.2 mCi/kg (3.7-7.4 MBq/kg) of ^18^F-FDG.

Trunk PET scans were performed from the upper thigh to the pharynx nasalis immediately after completion of the CT scans. After a skull CT scan, a 5-minute skull PET scan in one bed position was performed from the foramen magnum to the top of the skull. PET scans in center 1 and center 2 were respectively performed using a three-dimensional model with a matrix of 128×128 voxels and a two-dimensional model with a matrix of 128×128 voxels.

For CT reconstruction, the raw data were converted into images using two-dimensional fast Fourier transform. For three-dimensional reconstruction, CT data were converted by the digital model and prepared for attenuation correction of PET images by using voxel space overlay and interpolation. PET images were reconstructed using the iterative ordered-subset expectation maximization (OSEM) method (4 iteration times and 8 character sets) with scatter correction. Image fusion was completed, producing PET, CT, and PET/CT fusion images in the transverse, sagittal, and coronal planes.

### Tumor Segmentation

The contour of primary lung tumor (also called region of interest, ROI) on PET and CT images was first delineated slice-by-slice by a nuclear medicine physician with 10 years of experience in thoracic oncology using ITK-SNAP software (version 3.6.0; www.itksnap.org). To evaluate the reproducibility of feature extraction to different contours of segmentation, the delineated ROIs were perturbed by supervoxel–based contour randomization (24) to produce perturbed ROI in 60 randomly selected patients from training cohort.

### Radiomics Feature Extraction

Both PET and CT radiomics features were extracted using the PyRadiomics package (version 3.0; https://github.com/Radiomics/pyradiomics), an open-source platform for easy and reproducible radiomics feature extraction (25). 14 volumetric shape were separately extracted from PET and CT segmentation masks; 18 first-order statistical and 75 texture-matrix radiomics features were extracted from the original and 8 wavelet- and 2 Laplacian of Gaussian (LoG)-filtering-derived PET and CT images, respectively. Ultimately, 2074 = 2 modalities × [14 shape + (18 intensity+ 75 texture) × (1 + 8+2 images)] 3D radiomics features were extracted to quantify the phenotypic characteristics of the tumor region in PET and CT images. The parameter settings of PET/CT image preprocessing and the generation of derived images for customizing the PyRadiomics feature extraction are described in **Supplemental Table S1**. The comprehensive radiomics features list is described in **Supplemental Table S2**. To correct for differences in features caused by the different centers (i.e., different scanners), we used the ComBat compensation method (https://github.com/Jfortin1/ComBatHarmonization), which identified a center-specific transformation to express the feature data in a common space devoid of center effect (26–28). To further ensure that the range of features was relatively uniform, we firstly normalized the training data into z-scores, then the mean and standard deviations of training cohort were used to normalize the feature values of the internal and external validation cohorts.

### Radiomics Feature Selection, RAD-Score Building, and Prognostic Modeling

In order to avoid overfitting due to the over-abundance of features relative to sample size, we performed a four-step feature selection method to enable derivation of stable and effective signature in the training cohort. First, the two-way random effects, absolute agreement, single rater/measurement intra-class correlation coefficient (ICC) (29) was calculated for each feature to evaluate the robustness of feature extraction to different contours of segmentation. Features with ICC greater than 0.8 were considered as robust features and retained (30). Secondly, Pearson correlation analysis was used to assess the correlation between all remaining features. For each feature pair with absolute correlation coefficient > 0.8, the feature that yielded higher absolute column-wise correlation mean was eliminated, which tends to provide redundant information about tumor phenotype. Thirdly, univariate Cox analysis was conducted by performing 10-fold cross validation in the training cohort, and Harrell’s concordance index (C-index) was used to measure the prognostic performance. Features were sorted in descending order of the mean validation C-index (in the 10 validation rounds), and the top 10 features were selected. Finally, multivariate Cox analysis that considered all possible combinations of candidate features (in sets of 2 up to 10 features) was conducted by performing 10-fold cross validation in the training cohort, and the feature combination with the highest average validation C-index (in the 10-fold cross validation) and significantly associated with PFS was identified as prognostic signature. The signature was used to refit the final Cox model (Radiomics model) in the whole training cohort, and the radiomics signature score (Rad-score) for each patient was calculated based on the selected signature.

For clinicopathological parameters, univariate Cox analysis was performed to assess the association with PFS, and only significant predictors (*p* < 0.05; *p* values were corrected for false discovery rate (FDR)) after multiple testing correction with the Benjamini-Hochgerg method (31) in the training cohort, were kept to build multivariable Cox model (Clinicopathological model). To further assess whether the clinicopathological findings can improve the performance of the Rad-score, statistically significant clinicopathological parameters were combined with the Rad-score *via* forward stepwise feature selection using maximum log-likelihood criterion as the stopping rule (32), to build a combined multivariable Cox model (Combined model).

### Validation of Prognostic Model for PFS Prediction

Time-dependent receiver operating characteristic (ROC) analyses were performed to estimate the probability of each model in predicting 10-month, one-year and 14-month PFS. C-index and area under the ROC curve (AUC) were quantified to evaluate the prognostic accuracy in the training cohort and another two independent validation cohorts. The prognostic score was generated for multivariable Cox model by using a linear combination of selected features weighted by their respective coefficients. Patients were stratified into slow and rapid-progression subgroups by the median value of prognostic score as computed in training cohort; log-rank test was then used to compare the significant difference between the two Kaplan–Meier curves. The same median value of prognostic score was applied to the two independent validation cohorts to perform risk stratification. All patients who received EGFR-TKI therapy were therefore stratified into slow and rapid-progression subgroups. The PFS behaviors of the two subgroups were compared by Kaplan–Meier survival analysis.

### Statistical Analysis

Statistical analysis was performed with MATLAB R2015b, Statistical Program for Social Science (SPSS; version 22.0) and R software (version 3.4.4; http://www.Rproject.org). A two-sided *p* value < 0.05 was used as the criterion to indicate a statistically significant difference.

## Results

### Patient Characteristics

The baseline characteristics of the three patient cohorts with stage IIIC-IV EGFR-mutant NSCLC from two institutions are summarized in **Table 1**. 250 patients received first and second generation EGFR-TKI therapy (140 patients, 60 patients, and 50 patients in three cohorts), and 243 of the 250 (97.2%) patients suffered NSCLC progression during the follow-up period. The mean PFS for the training, internal validation, and external validation cohorts was 9.82 ± 6.86 months (range 0.3-35 months), 12.14 ± 8.47 months (range 1-40.5 months), and 15.20 ± 11.49 months (range 2.1-58.5 months), respectively.

**Table 1:** Demographic and clinicopathologic characteristics of the training cohort, internal validation cohort and external validation cohort.

Characteristics	Training set (n = 140)	Internal validation set (n = 60)	*p* value	External validation set (n = 50)
Age (years)			0.642	
Mean ± SD	57.83 ± 11.47	58.57 ± 12.25		61.46 ± 12.99
Gender			0.241	
Male	71 (50.7%)	25 (41.7%)		23 (46.0%)
Female	69 (49.3%)	35 (58.3%)		27 (54.0%)
Location			0.826	
Left	56 (40.0%)	25 (41.7%)		28 (56.0%)
Other	84 (60.0%)	35 (58.3%)		22 (44.0%)
Pathological typing			0.479	
Adenocarcinoma	133 (95.5%)	59 (98.3%)		48 (96.0%)
Other	7 (5.0%)	1 (1.7%)		2 (4.0%)
T category			0.925	
TI	22 (15.7%)	10 (16.7%)		6 (12.0%)
T2	49 (35.0%)	18 (30.0%)		12 (24.0%)
T3	28 (20.0%)	13 (21.7%)		6 (12.0%)
T4	41 (29.3%)	19 (31.7%)		26 (52.0%)
N category			0.454	
N0	15 (10.7%)	8 (13.3%)		9 (18.0%)
N1	20 (14.3%)	6 (1.0%)		3 (6.0%)
N2	51 (36.4%)	17 (28.3%)		6 (12.0%)
N3	54 (38.6%)	29 (48.3%)		32 (64.0%)
M category			0.231	
M0	5 (3.6%)	0 (0.0%)		2 (4.0%)
M1a	30 (21.4%)	16 (26.7%)		9 (18.0%)
M1b	60 (42.9%)	27 (45.0%)		10 (20.0%)
M1c	45 (32.1%)	17 (28.3%)		29 (58.0%)
Tobacco use			0.311	
Smoker	40 (28.6%)	13 (21.7%)		14 (28.0%)
No smoker	100 (71.4%)	47 (78.3%)		36 (72.0%)
Base PS score			0.770	
< 2	129 (92.1%)	56 (93.3%)		38 (76.0%)
≥ 2	11 (7.9%)	4 (6.7%)		12 (24.0%)
Mutation status			0.319	
EGFR 19Del	64 (45.7%)	30 (50.0%)		–
EGFR 21L858R	53 (37.9%)	25 (41.7%)		–
Other EGFR	23 (16.4%)	5 (8.3%)		–
CEA			0.835	
Mean ± SD	110.64 ± 209.31	130.37 ± 248.97		–
CYFRA21-1			0.987	
Mean ± SD	8.44 ± 11.37	10.19 ± 18.53		–
Brain metastasis			0.064	
Yes	19 (13.6%)	15 (25.0%)		11 (22.0%)
No	121 (86.4%)	45 (75.0%)		39 (78.0%)
Bone metastasis			0.275	
Yes	84 (60.0%)	31 (51.7%)		33 (66.0%)
No	56 (40.0%)	29 (48.3%)		17 (34.0%)
Liver metastasis			0.162	
Yes	21 (15.0%)	4 (6.7%)		2 (4.0%)
No	119 (85.0%)	56 (93.3%)		48 (96.0%)
Lung metastasis			0.828	
Yes	63 (45.0%)	28 (46.7%)		23 (46.0%)
No	77 (55.0%)	32 (53.3%)		27 (54.0%)
Pleural metastasis			0.225	
Yes	48 (34.3%)	26 (43.3%)		17 (34.0%)
No	92 (65.7%)	34 (56.7%)		33 (66.0%)
Adrenal metastasis			0.159	
Yes	25 (17.9%)	6 (10.0%)		10 (20.0%)
No	115 (82.1%)	54 (90.0%)		40 (80.0%)
PFS (months)			0.063	
Mean ± SD	9.82 ± 6.86	12.14 ± 8.47		15.20 ± 11.49

Age, CEA, CYFRA21-1 and PFS are shown as mean ± standard deviation (SD); other data are the number of patients with the percentage in parentheses. Statistical comparison between the training cohort and validation cohort was computed with χ2 test (categorical variables) or Mann-Whitney U test (continuous variables). PS, performance status; CEA, carcinoembryonic antigen; CYFRA21-1, non-small cell associated antigens; PFS, progression-free survival.

### PFS Prediction Performance of the RAD-Score and the Combined Model

A three-feature signature was built by four-step feature selection in the training cohort (**Supplemental Tables S3**, **S4**), and it was further used for the construction of multivariable radiomics model (**Supplemental Table S5**), and the Rad-score was calculated for each patient. Rad-score = 0.242 × PET_wavelet_LHL_First-order 90th percentile + 0.214 × PET_wavelet_LHL_GLDM Large Dependence High Gray Level Emphasis + 0.199 × CT_wavelet_HHH_First-order Kurtosis.

**Figure 1:**
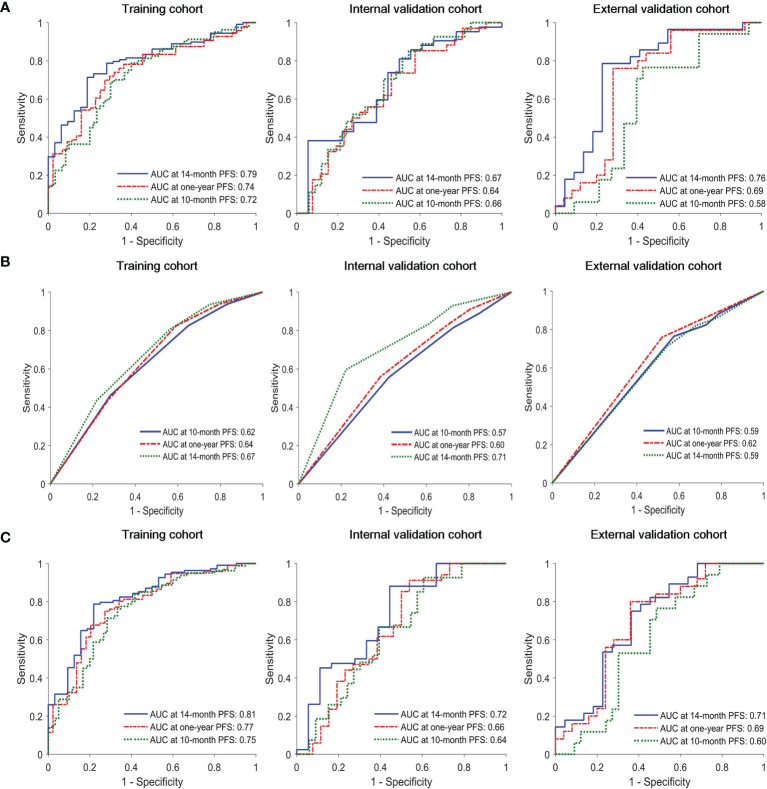
Time-dependent receiver operating characteristic (ROC) curves of **(A)** radiomics model, **(B)** clinical model and **(C)** combined model in the training, interval validation, and external validation cohorts.

The C-index of the radiomics model (Rad-score) for PFS prediction was 0.65 (95% confidence interval (CI): 0.60-0.70) for the training cohort and 0.61 (95% CI: 0.53-0.68) and 0.60 (95% CI: 0.52-0.68) for the internal and external validation cohorts, respectively (**Table 2**). The AUC for the radiomics model (Rad-score) ranged from 0.58 to 0.72, 0.64 to 0.74, and 0.67 to 0.79 for 10-month, one-year, and 14-month PFS probability prediction in the three cohorts, respectively (**Table 2** and **Figure 1A**).

**Table 2:** Model performance on predicting PFS and time-dependent PFS probability.

Model	Cohorts	C-index (95% CI)	AUC^1^ (95% CI)	AUC^2^ (95% CI)	AUC^3^ (95% CI)
**N stage^4^**	training	0.59 (0.54-0.64)	0.62 (0.54-0.71)	0.64 (0.55-0.72)	0.67 (0.58-0.74)
	internal validation	0.56 (0.47-0.64)	0.57 (0.44-0.70)	0.60 (0.47-0.73)	0.71 (0.58-0.82)
	external validation	0.56 (0.48-0.64)	0.59 (0.44-0.73)	0.62 (0.47-0.75)	0.59 (0.44-0.72)
**Rad-score**	training	0.65 (0.60-0.70)	0.72 (0.64-0.80)	0.74 (0.66-0.81)	0.79 (0.71-0.85)
	internal validation	0.61 (0.53-0.68)	0.66 (0.53-0.78)	0.64 (0.50-0.76)	0.67 (0.54-0.79)
	external validation	0.60 (0.52-0.68)	0.58 (0.43-0.72)	0.69 (0.55-0.81)	0.76 (0.62-0.87)
**Combined model**	training	0.67 (0.62-0.72)	0.75 (0.67-0.82)	0.78 (0.70-0.84)	0.81 (0.74-0.87)
	internal validation	0.61 (0.52-0.69)	0.64 (0.50-0.76)	0.66 (0.52-0.77)	0.72 (0.59-0.83)
	external validation	0.60 (0.51-0.69)	0.60 (0.45-0.73)	0.69 (0.55-0.81)	0.71 (0.56-0.83)

AUC^1^, AUCs at 10-month progression-free survival (PFS).

AUC^2^, AUCs at one-year progression-free survival (PFS).

AUC^3^, AUCs at 14-month progression-free survival (PFS).

N stage^4^, Clinicopathological model was built with N stage.

CI, confidence interval; AUC, area under the curve.

In univariate Cox proportional hazards regression analysis, after FDR correction, only N stage was found to be significantly associated with PFS in the training cohort. The corresponding C-index, *p* values and hazard ratios (HRs) with 95% CIs are detailed in **Supplemental Table S6**. The C-index of the clinical model (N stage) was 0.59 (95% CI: 0.54-64), 0.56 (95% CI: 0.47-0.64), and 0.56 (95% CI: 0.48-0.64) for training, internal and external validation cohorts, respectively. The AUC of clinical model for 10-month, one-year, 14-month PFS probability prediction ranged from 0.62-0.67, 0.57-0.71. and 0.59-0.62 in the three cohorts, respectively (**Table 2** and **Figure 1B**). The combined model incorporating the Rad-score and N stage yielded a C-index of 0.67 (95% CI: 0.62-0.72) in the training cohort, 0.61 (95% CI: 0.52-0.69) in the internal validation cohort, and 0.60 (95% CI: 0.51-0.69) in the external validation cohort; the AUC ranges were 0.60-0.75, 0.66-0.78, and 0.71-0.81 for the 10-month, one-year, and 14-month PFS probability predictions in the three cohorts, respectively (**Table 2** and **Figure 1C**). The statistical comparison of the ROC curves between the combined model and radiomics model (Rad-score) was performed using the DeLong test method (33). The *p* values from the DeLong test are given in **Supplemental Table S7**. No significant differences were found (all *p* values > 0.05); therefore, the combined model showed no performance improvement when compared with the radiomics model (Rad-score) for PFS prediction.

### Risk Stratification of EGFR-TKI Therapy and Identification of a Sensitive Population

**Figure 2:**
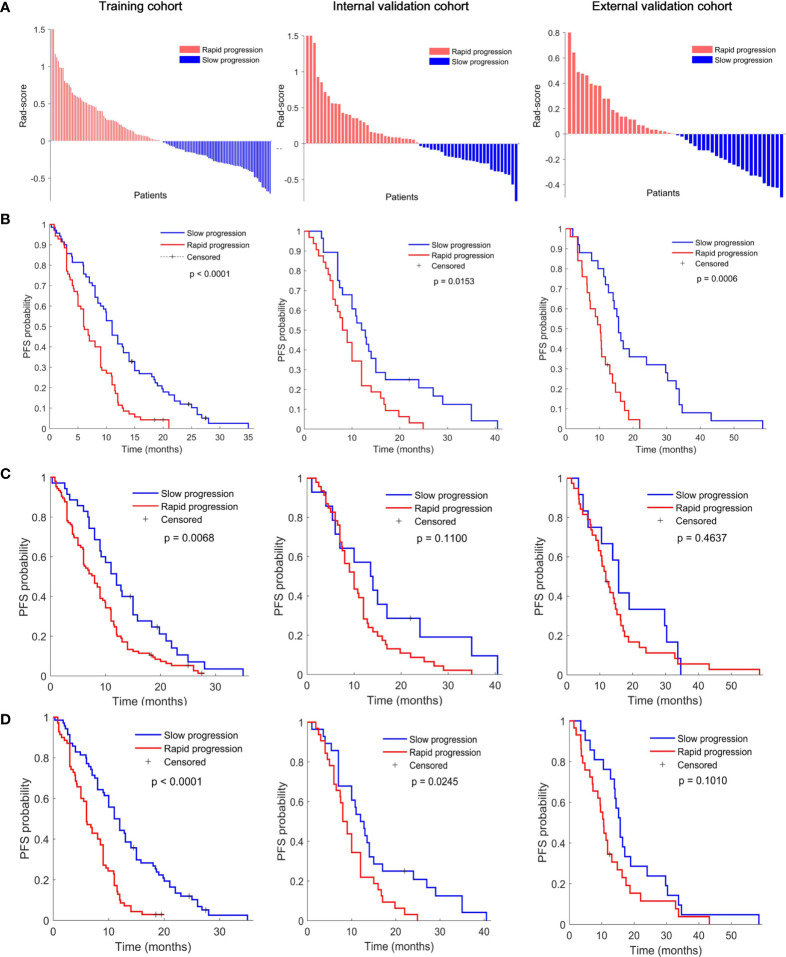
**(A)** Rad-score according to the three-feature signature and Kaplan–Meier survival curves of **(B)** radiomics model, **(C)** clinical model, and **(D)** combined model in the training (left), interval validation (middle), and external validation cohorts (right). All scores have subtracted the cutoff. *P* values were calculated using the log-rank test.

As shown in **Figures 2A, B** according to the division of median value of Rad-score (median value: -0.0721), each of these three cohorts was divided into slow-progression (blue bars) and rapid-progression (red bars) in the expectation of EGFR-TKI therapy. The Kaplan–Meier survival curves of the three cohorts present significant differences in PFS between the stratified slow and rapid progression subgroups. Higher Rad-scores were significantly associated with worse PFS in the training (*p* < 0.0001), internal validation (*p* = 0.0153), and external validation (*p* = 0.0006) cohorts. The proportion of rapidly progressing patients in each cohort was 50%, 47%, and 50%. Compared with the Rad-score, the Clinicopathological model based on N stage cannot identify the patients with a high risk of rapid progression in the internal (*p* = 0.1100) and external (*p* = 0.4637) validation cohorts (**Figure 2C**). At the same time, the combined model incorporating the Rad-score and N stage cannot successfully achieve risk stratification in the external (*p* = 0.1010) validation cohort (**Figure 2D**).

## Discussion

Our 2-center study results showed that the established PET/CT Rad-score had favorable predictive performance for PFS estimation. It can accurately quantify the risk of progression after first-line EGFR-TKI treatment in NSCLC patients and identify populations sensitive to targeted therapy for EGFR-mutant NSCLC to help develop personalized clinical treatment regimens.

At present, few studies based on PET/CT radiomics to predict PFS of NSCLC patients with EGFR mutations. Although, our previous study (23) attempted to investigate the performance of PFS prediction using interim PET/CT (∆SUVmax and ∆SUVmean) in stage IIIC/IV EGFR-mutant NSCLC patients with EGFR-TKI therapy, this is a single-centre study with a small cohort size (78 patients), the AUCs of the ∆SUVmax and ∆SUVmean were 0.764 and 0.725, respectively. This results has not been validated with independent cohorts from different centers, hence it may be over-fitting. Our results showed that the AUCs of the Rad-score were 0.72, 0.74, and 0.79 for 10-month, one-year, and 14-month PFS probability prediction in the training cohort. This is comparable with previous study. However, the corresponding AUCs were 0.66, 0.64 and 0.67 in the internal validation cohort, and 0.58, 0.69 and 0.76 in the external validation cohort, which reiterates the importance of multi-center independent validation to reflect the actual performance of radiomics model. Furthermore, this results are consistent with study by Kirienko et al. (15), which used PET/CT radiomics signatures to successfully predict disease-free survival (DFS) of 259 patients with NSCLC after surgery; it achieved an AUC of 0.68 *via* using PET/CT signature in the independent validation cohort (90 patients), and an AUC of 0.65 after combining it with clinical predictors. While the C-index of above studies were not reported, so there is no direct comparison of C-index. Importantly, our Rad-score had higher C-index and AUCs than clinical N stage in all three cohorts (**Table 2**), and can effectively identify the patients with low risk of slow progression in internal and external validation cohorts, and for these patients, EGFR-TKI therapy showed great clinical benefits (**Figure 2**).

In this study, a total of 2074 (1037 PET and 1037 CT) radiomics features were extracted to quantify the phenotypic characteristics of the tumor region in PET and CT images, and a 4-step feature selection method was performed to avoid model overfitting. In particular, the robustness of feature extraction to different contours of segmentation was evaluated, which may improve the stability and generalizability of model. Herein, we used supervoxel-based contour randomization (24) to create perturbed ROIs in PET and CT images of 60 randomly selected patients, respectively. Having 60 patients (120 PET and CT ROIs) perturbed would have made it possible to assess the robustness of feature extraction (34–36). Furthermore, due to the perturbed ROIs may deviate from tumor region, even include some non-tumor slices (**Supplementary Figure S1**), which may leads to unnecessary removal of features in robustness assessment, the perturbed results were further checked and adjusted slice by slice, which is also a time-consuming process.

**Figure 3:**
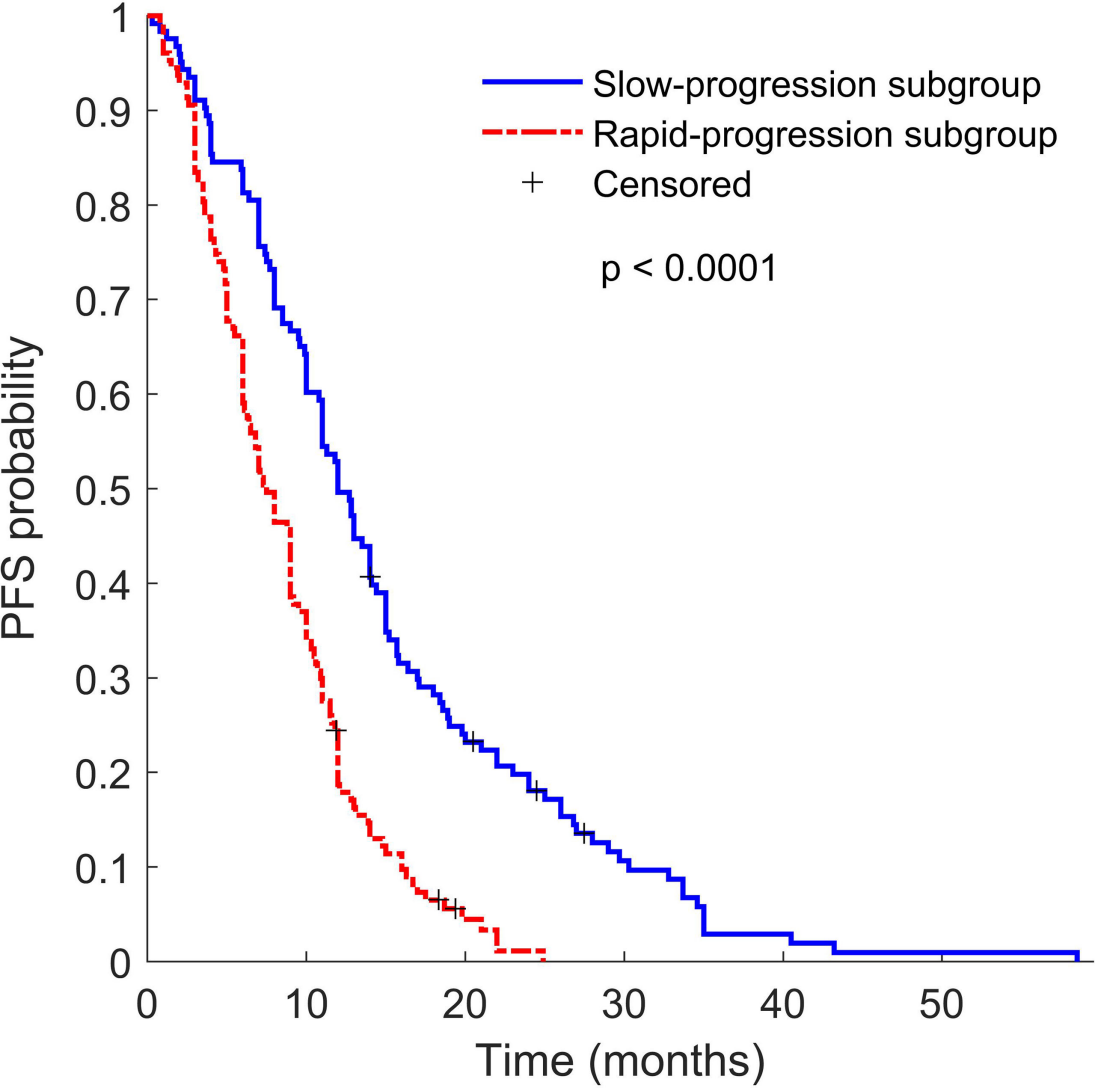
Kaplan–Meier survival curves of slow progression subgroup TKI patients (blue line) and rapid progression subgroup TKI patients (red line). *P* value was calculated using the log-rank test.

It is worth noting that nearly 90% (1867 of 2074) features were non-robust (309 of 2074 features) and redundant (1558 of 2074 features) (**Table S3**). Furthermore, Chalkidou et al. reported that 10 to 15 observations per predictor variable are minimally required to produce reasonably stable estimates (37). The top 10 features (140 training samples) were therefore selected from the remaining 10% features *via* univariate Cox analysis, and three-feature signature was eventually built *via* exhaustive search, which considered all possible combinations $\left(1013\text{ combinations}=\sum_{\text{k}=2}^{10}C_{10}^{k}\right)$ in multivariable Cox analysis. The signature consists of two PET features and one CT feature, namely PET_wavelet-LHL_first-order 90th Percentile (a measure to describe the 90th percentile of intensity distribution of wavelet-filtering PET images), PET_wavelet-LHL_gldm Large Dependence High Gray Level Emphasis (a measure to gauge the distribution of large dependence with higher gray-level values in Gray Level Dependence Matrix (GLDM) of wavelet-filtering PET images), and CT_wavelet-HHH_first order Kurtosis (a measure of peakedness in the intensity distribution of wavelet-filtering CT images). Our study showed that the Rad-score built with the three features could predict posttreatment PFS in the training group and the 2 validation groups of EGFR-TKI-treated patients (**Figure 2B**). The PET/CT Rad-score identified 123 patients with rapid progression after treatment and 127 patients with slow progression after treatment from 250 patients in the EGFR-TKI treatment group. The PFS of the corresponding two types of patients was 8.56 ± 5.17 months and 14.54 ± 10.19 months. There was a statistically significant difference (*p* < 0.0001) in PFS between the two groups (**Figure 3**). The rapid-progression patients in the EGFR-mutated NSCLC group did not benefit from EGFR-TKI treatment and were a TKI-treatment insensitive population. In this study, the PET/CT Rad-score successfully achieved accurate quantification of the risk of progression after first-line first and second generation EGFR-TKI drug therapy in stage IIIC/IV EGFR-mutated NSCLC patients and identified first and second generation EGFR-TKI treatment-sensitive populations.

Clinicopathological information on the combination of peripheral invasion and metastasis might reflect the biological invasiveness of tumors and have some predictive prognostic value (38). However, our survival analysis results identified only N stage among the clinicopathological factors as a risk factor for predicting PFS in patients receiving EGFR-TKI treatment, while lymph node metastasis is a well-established prognostic feature for predicting the prognosis of NSCLC (39–42). The predictive value of T stage and distant metastasis varies in different studies (4, 43–45), and our study showed that T stage, M stage, and metastasis at all sites were not prognostic factors. Smoking history is a causative risk factor for lung cancer (46), but whether smoking history is a risk factor affecting the therapeutic effect of lung cancer remains inconclusive (47–49). Pathological type and EGFR mutation (exon 18 deletion, exon 19 deletion, or exon 21 L858R substitution) subtype are still controversial prognostic factors in different trials (5, 39, 50–52). In our study, we found that differences in pathological type as well as three common EGFR mutations did not differ in terms of their benefit for EGFR-TKIs (*p* > 0.05). CEA and NSCLC-associated antigen are important indicators for monitoring the recurrence and progression of NSCLC, but our study did not show them as prognostic factors for predicting PFS. In addition, this analysis did not show that sex had a significant prognostic impact (41).

In the evaluation of the value of the clinicopathological model and Rad-score-clinicopathological combined model, our study showed that the combined model showed no significant PFS prediction performance improvement when compared with the Rad-score, and the AUC did not significantly improve. In addition, compared with the Rad-score, the clinicopathological model based on N stage had poor performance of risk stratification and could not identify sensitive patients with EGFR-TKI therapy in the internal (*p* = 0.1100) and external (*p* = 0.4637) validation cohorts. At the same time, the combined model incorporating the Rad-score and N stage could not successfully achieve risk stratification in the external validation cohort (*p* = 0.1010) and thus could not identify patients with rapid progression who are resistant to EGFR-TKI therapy. The possible reason may be that the clinical factor contains limited and rough prognostic information; moreover, due to tumor heterogeneity, pathological factors cannot reflect the overall tumor differentiation (53, 54). Our findings are in line with those of recent studies that the Rad-score-clinicopathological combined model does not improve the PFS prediction performance (15, 55).

Despite the favorable results of the Rad-score, our study also has some limitations. First, the PET/CT images from two centers were affected by different scanners and protocols. This effect was compensated by the ComBat harmonization method. A more comprehensive method to balance the scanner variance is worth future exploration. Second, we mined 3 prognostic features from PET/CT images and compared their performance with clinicopathological factors. The relationship between the radiomic features and biological level events was not investigated, which limits the broad translation of such radiomics model into clinical application.

Some efforts have been made to introduce biological meaning into radiomics. Tunali et al. performed genomic analysis and immunohistochemistry analysis for carbonic anhydrase IX, and demonstrated that CT treatment response biomarkers for patients with lung cancer treated with immunotherapy were strongly associated with hypoxia, a prognostic factor (56). Ganeshan et al. took a more detailed look at the correlation of CT texture features from lung cancer tumors to histopathologic markers of angiogenesis and hypoxia, and concluded that CT textures appear to be surrogate measure of tumor hypoxia (57). The interpretability of the radiomics model in terms of the biological properties of tissue is an essential and challenge process (58). Our future studies will plan to pursue biological validation to further explain the radiomics prognostic model.

## Conclusions

In conclusion, a pretreatment PET/CT radiomics biomarker for the prediction of the PFS of NSCLC patients with stage IIIC/IV EGFR mutations was established; it has the potential to realize the precise quantitative stratification of progression risk after first-line first and second generation of EGFR-TKI drug therapy and identify EGFR-mutant NSCLC populations sensitive to targeted therapy. One of the limitation is that the relationship between radiomics biomarker and biological meaning has not been investigated in present study.

## Data Availability Statement

The significant feature data supporting the conclusions of this article has been made publicly available. This data can be found here: https://github.com/dudongyangsmu/PFS-of-EGFR-mutated-NSCLC.

## Ethics Statement

The studies involving human participants were reviewed and approved by Research Ethics Committee, Guangdong Provincial People’s Hospital, Guangdong Academy of Medical Sciences. The ethics committee waived the requirement of written informed consent for participation. Written informed consent was not obtained from the individual(s) for the publication of any potentially identifiable images or data included in this article.

## Author Contributions

All authors listed have made a substantial, direct and intellectual contribution to the work, and approved it for publication.

## Funding

This work was supported by the National Natural Science Foundation of China under Grant 81871437, the NSFC Incubation Program of GDPH (KY012021162), the Science and Technology Planning Project of Guangdong Province (2019A1515011104, 2018A030313534, 2017A020215110), the Natural Science Foundation of Guangdong Province (2019A1515110377, 2018A030313534), the Guangdong Province Universities and Colleges Pearl River Scholar Funded Scheme (Lijun Lu, 2018), and the China Postdoctoral Science Foundation funded project under Grant 2020M682792.

## Conflict of Interest

The authors declare that the research was conducted in the absence of any commercial or financial relationships that could be construed as a potential conflict of interest.

## Publisher’s Note

All claims expressed in this article are solely those of the authors and do not necessarily represent those of their affiliated organizations, or those of the publisher, the editors and the reviewers. Any product that may be evaluated in this article, or claim that may be made by its manufacturer, is not guaranteed or endorsed by the publisher.
