# Supplementary material for: Identification of Stage IIIC/IV EGFR-Mutated Non-Small Cell Lung Cancer Populations Sensitive to Targeted Therapy Based on a PET/CT Radiomics Risk Model
Source: Front Oncol. 2021 Nov 2;11:721318. doi: 10.3389/fonc.2021.721318 (PMC8593197; doi:10.3389/fonc.2021.721318)
Supplement: Supplementary file 1 [file Table_1.docx]

**Supplementary Tables**

**Table S1.** The detailed description of PET/CT image preprocessing and generation of derived images for customizing extraction in Pyradiomics.

| **PET/CT image preprocessing** | | |
| --- | --- | --- |
| To mitigate the effects of different voxel sizes (accounting for the pixel size and slice spacing) on radiomics features [1], and guarantee rotational invariance of texture features [2], isotropic resampling was required to allow comparison between image data from different samples, cohorts or batches. In addition, discretization processing of images is used for texture and certain first-order features computation and image noise reduction [2]. Appropriate bin widths [3] were used to avoid over or under binning an image, as follows: | | |
| Parameter | PET | CT |
| Interpolation |  |  |
| Interpolation method | trilinear | trilinear |
| Resampled voxel spacing | 4×4×4 mm | 2×2×2 mm |
| Discretization |  |  |
| Discretization method | FBS | FBS |
| Bin size | 0.1 SUV | 25 HU |
| **Generation of derived images** | | |
| To enhance specific imaging characteristics, wavelet and Laplacian of Gaussian (LOG) filters were applied to original PET and CT images, yield corresponding derived images [4]. A ‘coif1’ wavelet transform was applied to each CT or PET image, which decomposes the original image into 8 decompositions by performing low-pass or high-pass filtering in *x*-, *y*-, or *z*-directions. Edge-enhancement LoG filter was also applied to each CT or PET image, which emphasizes areas of gray level change, and defines how coarse the emphasized texture should be via the width of the filter in the Gaussian kernel (sigma). A low sigma value emphasizes fine textures (gray level change over a short distance), while a high sigma value emphasizes coarse textures (gray level change over a large distance). | | |
| Parameter | PET | CT |
| wavelet | coif1 | coif1 |
| sigma | 4 mm, 8 mm | 2 mm, 4 mm |

**Table S2.** List of extracted radiomics features.

| **Feature Class (Extraction description)** | | **Feature name** |
| --- | --- | --- |
| **Shape** (The shape features were only extracted from original PET and CT images.) | 1 | Elongation |
|  | 2 | Flatness |
|  | 3 | Least Axis Length |
|  | 4 | Major Axis Length |
|  | 5 | Maximum 2D Diameter (Column) |
|  | 6 | Maximum 2D Diameter (Row) |
|  | 7 | Maximum 2D Diameter (Slice) |
|  | 8 | Maximum 3D Diameter |
|  | 9 | Mesh Volume |
|  | 10 | Minor Axis Length |
|  | 11 | Sphericity |
|  | 12 | Surface Area |
|  | 13 | Surface Area to Volume Ratio |
|  | 14 | Voxel Volume |
| **First-order** (The first-order statistical features were extracted from original, wavelet-filtering, and LOG-filtering PET and CT images.) | 1 | 10th percentile |
|  | 2 | 90th percentile |
|  | 3 | Energy |
|  | 4 | Entropy |
|  | 5 | Interquartile Range |
|  | 6 | Kurtosis |
|  | 7 | Maximum |
|  | 8 | Mean Absolute Deviation |
|  | 9 | Mean |
|  | 10 | Median |
|  | 11 | Minimum |
|  | 12 | Range |
|  | 13 | Robust Mean Absolute Deviation |
|  | 14 | Root Mean Squared |
|  | 15 | Skewness |
|  | 16 | Total Energy |
|  | 17 | Uniformity |
|  | 18 | Variance |
| **Texture - Gray Level Cooccurrence Matrix (GLCM) Features** (The GLCM features were extracted from original, wavelet-filtering, and LOG-filtering PET and CT images.) | 1 | Autocorrelation |
|  | 2 | Cluster Prominence |
|  | 3 | Cluster Shade |
|  | 4 | Cluster Tendency |
|  | 5 | Contrast |
|  | 6 | Correlation |
|  | 7 | Difference Average |
|  | 8 | Difference Entropy |
|  | 9 | Difference Variance |
|  | 10 | Inverse Difference |
|  | 11 | Inverse Difference Moment |
|  | 12 | Inverse Difference Moment Normalized |
|  | 13 | Inverse Difference Normalized |
|  | 14 | Informational Measure of Correlation 1 |
|  | 15 | Informational Measure of Correlation 2 |
|  | 16 | Inverse Variance |
|  | 17 | Joint Average |
|  | 18 | Joint Energy |
|  | 19 | Joint Entropy |
|  | 20 | Maximal Correlation Coefficient |
|  | 21 | Maximum Probability |
|  | 22 | Sum Average |
|  | 23 | Sum Entropy |
|  | 24 | Sum of Squares |
| **Texture - Gray Level Size Zone Matrix (GLSZM) Features** (The GLSZM features were extracted from original, wavelet-filtering, and LOG-filtering PET and CT images.) | 1 | Gray Level Non-Uniformity |
|  | 2 | Gray Level Non-Uniformity Normalized |
|  | 3 | Gray Level Variance |
|  | 4 | High Gray Level Zone Emphasis |
|  | 5 | Large Area Emphasis |
|  | 6 | Large Area High Gray Level Emphasis |
|  | 7 | Large Area Low Gray Level Emphasis |
|  | 8 | Low Gray Level Zone Emphasis |
|  | 9 | Size Zone Non-Uniformity |
|  | 10 | Size Zone Non-Uniformity Normalized |
|  | 11 | Small Area Emphasis |
|  | 12 | Small Area High Gray Level Emphasis |
|  | 13 | Small Area Low Gray Level Emphasis |
|  | 14 | Zone Entropy |
|  | 15 | Zone Percentage |
|  | 16 | Zone Variance |
| **Texture - Gray Level Run Length Matrix (GLRLM) Features** (The GLRLM features were extracted from original, wavelet-filtering, and LOG-filtering PET and CT images.) | 1 | Gray Level Non-Uniformity |
|  | 2 | Gray Level Non-Uniformity Normalized |
|  | 3 | Gray Level Variance |
|  | 4 | High Gray Level Run Emphasis |
|  | 5 | Long Run Emphasis |
|  | 6 | Long Run High Gray Level Emphasis |
|  | 7 | Long Run Low Gray Level Emphasis |
|  | 8 | Low Gray Level Run Emphasis |
|  | 9 | Run Entropy |
|  | 10 | Run Length Non-Uniformity |
|  | 11 | Run Length Non-Uniformity Normalized |
|  | 12 | Run Percentage |
|  | 13 | Run Variance |
|  | 14 | Short Run Emphasis |
|  | 15 | Short Run High Gray Level Emphasis |
|  | 16 | Short Run Low Gray Level Emphasis |
| **Texture - Neighboring Gray Tone Difference Matrix (NGTDM) Features** (The NGTDM features were extracted from original, wavelet-filtering, and LOG-filtering PET and CT images.) | 1 | Busyness |
|  | 2 | Coarseness |
|  | 3 | Complexity |
|  | 4 | Contrast |
|  | 5 | Strength |
| **Texture - Gray Level Dependence Matrix (GLDM) Features** (The GLDM features were extracted from original, wavelet-filtering, and LOG-filtering PET and CT images.) | 1 | Dependence Entropy |
|  | 2 | Dependence Non-Uniformity |
|  | 3 | Dependence Non-Uniformity Normalized |
|  | 4 | Dependence Variance |
|  | 5 | Gray Level Non-Uniformity |
|  | 6 | Gray Level Variance |
|  | 7 | High Gray Level Emphasis |
|  | 8 | Large Dependence Emphasis |
|  | 9 | Large Dependence High Gray Level Emphasis |
|  | 10 | Large Dependence Low Gray Level Emphasis |
|  | 11 | Low Gray Level Emphasis |
|  | 12 | Small Dependence Emphasis |
|  | 13 | Small Dependence High Gray Level Emphasis |
|  | 14 | Small Dependence Low Gray Level Emphasis |

Complete list of Pyradiomics [4] features used in this study. Exact feature definitions are provided in <https://pyradiomics.readthedocs.io/>.

**Table S3.** Number of features retained after each selection step in the training cohort.

| Features (PET/CT) | Robustness analysis (PET/CT) | Redundancy analysis (PET/CT) | Univariate Cox analysis (PET/CT) | Multivariate Cox analysis (PET/CT) |
| --- | --- | --- | --- | --- |
| 2074 (1037/1037) | 1765 (951/814) | 207 (88/119) | 10 (9/1) | 3 (2/1) |

**Table S4**. The C-index and *p* values of the top 10 features selected in the univariate Cox analysis.

| Feature | C-index | *p* value |
| --- | --- | --- |
| CT_wavelet-HHH_firstorder_Kurtosis | 0.6127 | 0.0001 |
| PET_wavelet-LHL_gldm_  LargeDependenceHighGrayLevelEmphasis | 0.6127 | 1.33 × 10^-6^ |
| PET_wavelet-LHL_glszm_  LargeAreaLowGrayLevelEmphasis | 0.6109 | 0.2631 |
| PET_log-sigma-4-0-mm-3D_glcm_JointEnergy | 0.6108 | 0.2397 |
| PET_wavelet-LLH_firstorder_Uniformity | 0.6032 | 0.0400 |
| PET_wavelet-LHL_gldm_  LargeDependenceLowGrayLevelEmphasis | 0.6030 | 0.0344 |
| PET_log-sigma-8-0-mm-3D_glcm_ClusterProminence | 0.6021 | 0.0023 |
| PET_wavelet-LHL_firstorder_90Percentile | 0.6009 | 0.0004 |
| PET_log-sigma-8-0-mm3D_glszm_  LargeAreaLowGrayLevelEmphasis | 0.5985 | 0.5496 |
| PET_log-sigma-8-0-mm-3D_gldm_LargeDependenceLowGrayLevelEmphasis | 0.5973 | 0.3720 |

**Table S5.** Results of radiomics feature selection in the training cohort.

| Feature | Coefficient | HR (95% CI) | *p-cox* |
| --- | --- | --- | --- |
| PET_wavelet-LHL_firstorder 90Percentile | 0.2419 | 1.274 (1.043-1.560) | 0.0195 |
| CT_wavelet-HHH_firstorder Kurtosis | 0.2144 | 1.239 (1.035-1.488) | 0.0215 |
| PET_wavelet-LHL_gldm LargeDependenceHighGrayLevelEmphasis | 0.1990 | 1.220 (1.003-1.488) | 0.0490 |

Abbreviations: HR, hazard ratio; CI, confidence interval; *p-cox*, the significance of regression coefficient in multivariate Cox regression model.

**Table S6.** Univariate association of demographic and clinicopathologic characteristics and progression-free survival in the training cohort.

| Feature | C-index | *p* value | HR (95 % CI) |
| --- | --- | --- | --- |
| Age | 0.57 | 0.5765 | 1.007 (0.992-1.023) |
| Gender | 0.52 | 0.4556 | 0.806 (0.569-1.141) |
| Tumor location | 0.52 | 0.2481 | 1.451 (1.004-2.096) |
| Pathological typing | 0.51 | 0.6535 | 1.353 (0.621-2.948) |
| T category | 0.53 | 0.4142 | 1.117 (0.952-1.311) |
| N category | 0.59 | 0.0301 | 1.329 (1.108-1.594) |
| M category | 0.54 | 0.4667 | 1.133 (0.913-1.404) |
| Tobacco use | 0.52 | 0.4051 | 1.350 (0.921-1.978) |
| Base PS score | 0.52 | 0.4142 | 1.608 (0.851-3.037) |
| Mutation status | 0.50 | 0.8317 | 0.955 (0.752-1.213) |
| CEA | 0.51 | 0.7462 | 1.000 (0.999-1.001) |
| CYFRA21-1 | 0.56 | 0.2481 | 1.014 (0.999-1.029) |
| Brain metastasis | 0.53 | 0.2481 | 1.626 (0.973-2.718) |
| Bone metastasis | 0.51 | 0.8317 | 0.963 (0.676-1.372) |
| Liver metastasis | 0.51 | 0.6535 | 1.197 (0.735-0.951) |
| Lung metastasis | 0.52 | 0.8317 | 0.943 (0.666-1.335) |
| Pleural metastasis | 0.50 | 0.8317 | 1.045 (0.723-1.509) |
| Adrenal metastasis | 0.52 | 0.4142 | 1.368 (0.866-2.163) |

Abbreviations: HR, hazard ratio; CI, confidence interval; PS, performance status; CEA, carcinoembryonic antigen; CYFRA21-1, non-small cell associated antigens.

**Table S7.** The *p* values of ROC comparison between Rad-score and combined model for 10 month, one-year, and 14-month PFS probability prediction in three cohorts.

| *P* value | 10-month PFS | one-year PFS | 14-month PFS |
| --- | --- | --- | --- |
| Training cohort | 0.26 | 0.26 | 0.49 |
| Internal validation cohort | 0.54 | 0.67 | 0.25 |
| External validation cohort | 0.81 | 0.98 | 0.33 |

The *p* values were calculated using Delong test [5].

Abbreviations: PFS, progression-free survival.


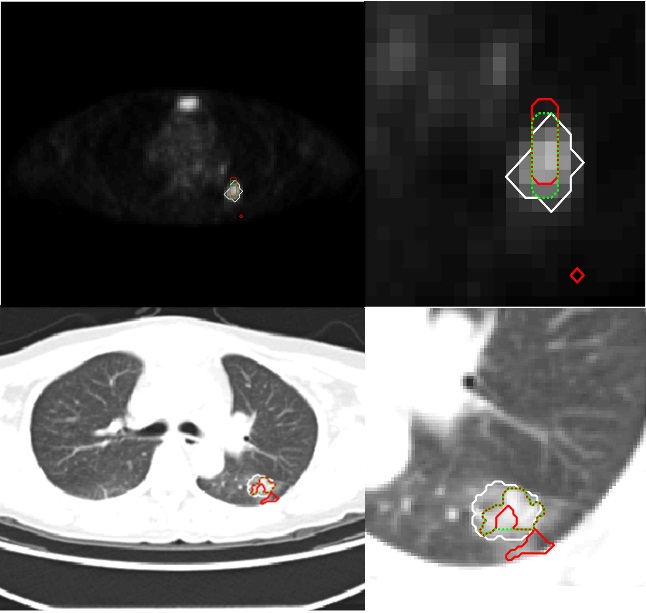


**Figure S1**. Example of PET and CT segmentations: (white line) original contour, (red line) perturbed contour, and (green line) adjusted contour after perturbation.

References

1. Ligero M, Jordi-Ollero O, Bernatowicz K, et al (2020) Minimizing acquisition-related radiomics variability by image resampling and batch effect correction to allow for large-scale data analysis. Eur Radiol. https://doi.org/10.1007/s00330-020-07174-0

2. Zwanenburg A, Leger S, Vallières M, Löck S (2016) Image biomarker standardisation initiative. ArXiv161207003 Cs

3. Du D, Gu J, Chen X, et al (2020) Integration of PET/CT Radiomics and Semantic Features for Differentiation between Active Pulmonary Tuberculosis and Lung Cancer. Mol Imaging Biol. https://doi.org/10.1007/s11307-020-01550-4

4. van Griethuysen JJM, Fedorov A, Parmar C, et al (2017) Computational Radiomics System to Decode the Radiographic Phenotype. Cancer Res 77:e104–e107. https://doi.org/10.1158/0008-5472.CAN-17-0339

5. DeLong ER, DeLong DM, Clarke-Pearson DL (1988) Comparing the Areas under Two or More Correlated Receiver Operating Characteristic Curves: A Nonparametric Approach. Biometrics 44:837-. https://doi.org/10.2307/2531595
